# Inhibition of hyaluronan retention by 4-methylumbelliferone suppresses osteosarcoma cells *in vitro* and lung metastasis *in vivo*

**DOI:** 10.1038/bjc.2011.459

**Published:** 2011-11-01

**Authors:** E Arai, Y Nishida, J Wasa, H Urakawa, L Zhuo, K Kimata, E Kozawa, N Futamura, N Ishiguro

**Affiliations:** 1 Department of Orthopedic Surgery, Nagoya University Graduate School of Medicine, 65-Tsurumai, Showa, Nagoya, Aichi 466-8550, Japan; 2 Research Complex for the Medicine Frontiers, Aichi Medical University, Karimata 21, Yazako, Nagakute, Aichi 480-1195, Japan

**Keywords:** 4-methylumbelliferone, hyaluronan, extracellular matrix, osteosarcoma, lung metastasis

## Abstract

**Background:**

Hyaluronan (HA) plays crucial roles in the tumourigenicity of many types of malignant tumours. 4-Methylumbelliferone (MU) is an inhibitor of HA synthesis. Several studies have shown its inhibitory effects on malignant tumours; however, none have focused on its effects on osteosarcoma.

**Methods:**

We investigated the effects of MU on HA accumulation and tumourigenicity of highly metastatic murine osteosarcoma cells (LM8) that have HA-rich cell-associated matrix, and human osteosarcoma cell lines (MG-63 and HOS).

**Results:**

*In vitro*, MU inhibited HA retention, thereby reducing the formation of functional cell-associated matrices, and also inhibited cell proliferation, migration, and invasion. Akt phosphorylation was suppressed by MU (1.0 mM). *In vivo*, although MU showed only a mild inhibitory effect on the growth of the primary tumour, it markedly inhibited (75% reduction) the development of lung metastasis. Hyaluronan retention in the periphery of the primary tumour was markedly suppressed by MU.

**Conclusion:**

These findings suggested that MU suppressed HA retention and cell-associated matrix formation in osteosarcoma cells, resulting in a reduction of tumourigenicity, including lung metastasis. 4-Methylumbelliferone is a promising therapeutic agent targeting both primary tumours and distant metastasis of osteosarcoma, possibly via suppression of HA retention.

The prognosis of osteosarcoma patients has improved dramatically with the introduction of chemotherapy; however, cases with metastases or an unresectable tumour still have a poor prognosis. Moreover, survival of patients with osteosarcoma has not improved with conventional chemotherapy over the past two decades. There is, thus, an urgent need to develop novel agents that do not cause serious complications or interfere with the function, ADL, or QOL of patients with osteosarcoma.

Increased hyaluronan (HA) levels in malignant tumours have been reported in cases of gastric, colorectal, breast, glioma, lung, and ovarian cancers (Pirinen *et al*, 1998; Ropponen *et al*, 1998; Setala *et al*, 1999; Anttila *et al*, 2000; Auvinen *et al*, 2000). Several studies have shown that HA levels correlate with the proliferation, motility, invasion, and metastasis of malignant tumour cells (Mummert *et al*, 2003; Ricciardelli *et al*, 2007; Bharadwaj *et al*, 2009). This may occur partly because an increased HA-rich matrix may support invasion by providing cancer cells with a suitable microenvironment (Knudson *et al*, 1989), stimulate cell motility via interactions of HA with cell-surface receptors (Yang *et al*, 1993), or form a barrier for cancer cells against host immunocompetent cells (McBride and Bard, 1979). Moreover, increased pericellular HA levels may lead to drug resistance against anticancer agents (Suzuki *et al*, 2009; Toole, 2009).

In osteosarcoma, antisense inhibition of HAS2 suppressed HA retention and tumourigenicity (Nishida *et al*, 2005), and administration of HA oligosaccharides inhibited endogenous HA deposition via interaction with CD44, a cell-surface receptor of HA, resulting in suppression of the tumourigenicity of two osteosarcoma cell lines (Hosono *et al*, 2007). However, the clinical application of these modalities is difficult because of ethical issues and/or the problems associated with efficient drug delivery.

4-Methylumbelliferone (MU), a modified coumarin (7-hydroxy-4-methylcoumarin), is a cholagogue and is widely used as an oral medicine in Japan (Takeda and Aburada, 1981). It is also known to inhibit HA synthesis via glucuronidation by endogenous glucuronosyltransferase (UGT), which results in depletion of uridine diphosphate glucuronic acid (UDP-GlcUA) (Kakizaki *et al*, 2004). Some studies have shown the anticancer effects of MU through the inhibition of HA synthesis *in vitro* and *in vivo* with or without anticancer agents (Yoshihara *et al*, 2005). However, no study has investigated the inhibitory effects of MU on osteosarcoma. Compared with epithelial malignant tumours, malignant cells of mesenchymal origin have more abundant ECM, and HA plays a crucial role in maintaining the integrity of ECM.

In this study, we hypothesised that the inhibition of HA synthesis by MU in osteosarcoma cells with an HA-rich ECM might alter the formation of ECM and the tumourigenicity of cells. We investigated the *in vitro* and *in vivo* effects of MU on highly metastatic osteosarcoma cells, LM8, that have an HA-rich ECM, in addition to the human osteosarcoma cell lines MG-63 and HOS.

## Materials and methods

### Chemicals

4-Methylumbelliferone was purchased from Wako Pure Chemicals (Osaka, Japan). Highly purified hyaluronan (Artz) was purchased from Kaken Pharmaceutical (Tokyo, Japan). Specific primers for mouse Has1 (hyaluronan synthase-1), Has2 (hyaluronan synthase-2), Has3 (hyaluronan synthase-3), CD44, Hyal1 (hyaluronidase-1), Hyal2 (hyaluronidase-2), Hyal3 (hyaluronidase-3), and glyceraldehyde-3-phosphate dehydrogenase (GAPDH) and human HAS1 (hyaluronan synthase-1), HAS2 (hyaluronan synthase-2), HAS3 (hyaluronan synthase-3), CD44, and GAPDH were custom made by Nihon Gene Research Laboratories (Sendai, Japan). Short-interfering RNAs (MISSION esiRNA) were purchased from Sigma-Aldrich (St Louis, MO, USA).

### Cell culture

All experiments were conducted according to the Regulations for Animal Experiments in our institution, and the Fundamental Guidelines for Proper Conduct of Animal Experiments and Related Activities in Academic Research Institutions in Japan. The mouse highly metastatic osteosarcoma cell line, LM8, was a kind gift from Mie University (Mie, Japan). The human osteosarcoma cell lines, MG-63 and HOS, were purchased from the American Type Culture Collection (Manassas, VA, USA). The cells were grown as monolayers. The MU stock solution for *in vitro* experiments was dissolved in DMSO, and the final concentration of DMSO in the medium was adjusted to 1.0%.

### Observation of functional cell-associated matrix

Cell-associated pericellular matrices were observed using a particle exclusion assay (Knudson, 1993). The cells were observed and photographed with an inverted phase-contrast microscope. The functional cell-associated matrix areas of randomly selected cells were captured as digital images and analysed using Scion Image software (Scion Corporation, Frederick, MD, USA). Morphometric analyses were performed to determine the proportions of the area delineated by the cell-associated matrix area to the area delineated by the plasma membrane area. To analyse whether exogenous HA is able to cancel the MU effects, cell-associated matrix formation was determined after a 72-h co-incubation of 1.0 mM MU with or without 200 *μ*g ml^–1^ of exogenous HA.

### Conventional and real time RT-PCR analysis

On the basis of a recent report that showed that MU downregulated HA mRNA levels in several human cell lines (Kultti *et al*, 2009), we determined the expression levels of Has1, Has2, Has3, and CD44 in LM8 cells after incubation with or without MU. Cells were cultured with or without 1.0 mM MU for 24 h. Total cellular RNA was isolated, and subjected to the conventional reverse transcriptase-PCR (RT-PCR). The RT-PCR revealed that Has2 mRNA could not be detected in LM8 cells, and cDNA was subjected to real-time RT-PCR for semiquantification of Has1, Has3, and CD44 mRNAs using a LightCycler (Roche Diagnostics, Mannheim, Germany). The relative levels of target mRNAs in a sample were expressed as relative quantification normalised against GAPDH mRNA levels. We also determined the mRNA expression of Hyal1, Hyal2, and Hyal3 in LM8 cells after 24 h of incubation with or without MU using the conventional RT-PCR. Expression levels for Bax and bcl-2 mRNA were determined using real-time RT-PCR for evaluation of apoptotic activity. Sequences of primer pairs used in this study, and expected product size are provided in Supplementary Online Material.

### Immunocytochemistry for Has

The LM8 cells were seeded onto chamber slides (BD Biosciences, Mountain View, CA, USA), allowed to adhere to the bottom of the slides for 12 h, and then incubated with 0–1.0 mM MU for 24 h. Then, cultured cells were subjected to Has1 and Has3 immunocytochemistry. Polyclonal antibodies against Has1 and Has3 were raised in rabbits by subcutaneous injection of the following synthetic peptides: VRRLCRRRSGGTRVGV, corresponding to amino acids 568–582 of Has1, and CGKKPEQYSLAFAEV, corresponding to amino acids 541–555 of Has3, which had been coupled to keyhole limpet haemacyanin. The specificity of the purified antibodies has been confirmed (Tominaga *et al*, 2001).

### Cell viability assay

Cultured cells were exposed to 10% FBS medium with 1% DMSO containing 0–1.0 mM MU with or without 200 *μ*g ml^–1^ of exogenous HA. A previous report showed that the molecular weight of HA synthesised by Has1 or Has2 was 2 × 10^5^ to ∼2 × 10^6^ Da, and that of Has3 was 1 × 10^5^ to 1 × 10^6^ Da (Itano *et al*, 1999). Considering that MU inhibits HA synthesised by Has1-3, we used ∼8 × 10^5^ Da exogenous HA within the range of HA molecular weight synthesised endogenously. After 24, 48, and 72 h of treatment, cell proliferation was measured using the 3-(4,5-dimethylthiazol-2-yl)-2,5,diphenyl tetrazolium bromide (MTT) colorimetric assay with Cell proliferation Kit I (Roche Diagnostics).

### TUNEL staining

LM8 cells were incubated with or without 1.0 mM MU for 48 h. The cells were fixed with paraformaldehyde and subjected to TUNEL (terminal deoxynucleotidyltransferase-mediated dUTP nick end labelling) stained using an *In Situ* Cell Death Detection Kit, POD (Roche Diagnostics). Cells with brown-stained nuclei in 10 different fields (200–300 cells per field) were counted under a light microscope at × 400 magnification, and the percentage of positively stained cells was calculated.

### Cell-cycle analysis

LM8 cells were incubated with or without 1.0 mM MU for 24 h. The cells were washed with phosphate-buffered saline (PBS), trypsinised, followed by permeabilisation and treatment with RNase, and stained with propidium iodide using a CycleTEST PLUS DNA reagent kit (BD Biosciences). The DNA content of the stained cells was immediately analysed using FACSCalibur (BD Biosciences). The percentages of cells in G0/G1 phase, S phase, and G2/M phase were determined using ModiFit LT software (Verity Software House, Topsham, ME, USA).

### Motility and matrigel invasion assays

The chemotactic motilities of cells were investigated using 12-well cell culture chambers containing inserts with 12-*μ*m pores (Millipore, Billerica, MA, USA). Invasion of LM8 cells was assayed in the same chambers that contained the inserts with 12-*μ*m pore membrane coated with matrigel. The cells were added to the upper chamber at a density of 5 × 10^5^ cells per insert in the presence or absence of 0.1–1.0 mM MU, and a chemotaxis buffer containing 10 *μ*g ml^–1^ of fibronectin was added to the lower chamber. After 24 h of incubation, cells on the upper surface were wiped off with a cotton swab. Cells that had invaded the lower surface were fixed with 70% ethanol and stained with haematoxylin. Cells from 10 different fields were counted under a light microscope at × 400 magnification.

In addition, to determine whether the effects of MU are mediated by inhibition of HA synthesis, cell motility and invasiveness with MU were evaluated after knockdown of HA synthases using siRNAs for Has1 and/or Has3. Briefly, siRNAs (MISSION esiRNA; Sigma-Aldrich) for Has1 and/or Has3 were complexed with Lipofectamine 2000 (Invitrogen, Carlsbad, CA, USA) in Opti-MEM (Invitrogen) according to the manufacturer's protocol, and then the mixtures were administered to LM8 cells in DMEM with serum and antibiotics. As a control siRNA, GFP siRNA was used. For 48-h incubation, efficiency of knockdown with siRNA for Has1 and/or Has3 was confirmed using real-time RT-PCR. The effects of MU on motility and invasiveness were analysed under the condition of Has1 and/or Has3 knockdown.

### Western blot analysis

The effects of MU on the expression levels of Akt and phospho-Akt (p-Akt) protein were assayed by western blot analysis. LM8 cells were incubated with or without 1.0 mM MU for 30 min, and 3, 6, and 12 h, and the cells were lysed on ice in RIPA buffer (Santa Cruz Biotechnology, Santa Cruz, CA, USA). After centrifugation at 10 000 g for 10 min, the supernatant was subjected to western blot analysis. Protein concentration was determined using the protein assay reagent (Bio-Rad, Philadelphia, PA, USA). The extracted protein (40 *μ*g per lane) was subjected to western blot analysis using rabbit anti-Akt and anti-phospho-Akt (Ser473) polyclonal antibodies (Cell Signaling Technology, Beverly, MA, USA). Species crossreactivity of these polyclonal antibodies with murine Akt and phospho-Akt has been already confirmed by the manufacturer (Cell Signaling Technology).

### Effects of MU *in vivo*

LM8 cells are known to be tumourigenic when injected subcutaneously into syngeneic hosts, and they consistently grow as local tumour masses and develop distant lung metastases (Asai *et al*, 1998). LM8 cells (2 × 10^6^) suspended in 200 *μ*l of serum-free DMEM were implanted into the dorsal flank of 5-week-old C3H/He male mice and allowed to grow *in vivo* for a period of 14 days, at which time small tumours (0.8–1.0 cm in diameter) were identified. The mice were randomly divided into two groups (*n*=8 mice per group). 4-Methylumbelliferone (10 mg per body) with 100 *μ*l of 0.4% CMC solution was intraperitoneally administered daily to mice in the MU group. The same amount of 0.4% CMC solution was administered to the mice in the control group. After 2 weeks of consecutive administration, all the mice were killed, and their local tumours and lungs were excised and analysed for local tumour wet weight and number of metastatic colonies in the lung. All animal experiments were performed in accordance with the National Cancer Research Institute (2010) Guidelines for the welfare and use of animals in cancer research (Workman *et al*, 2010) and under approval of the institutional animal ethics committee.

### HA staining for cells and tissues

Hyaluronan accumulation in cells and *in vivo* tissues after incubation with or without MU was observed using hyaluronic acid binding protein (HABP; Seikagaku, Tokyo, Japan). The cells were seeded onto chamber slides (BD Biosciences), allowed to adhere to the bottom of the slides for 12 h, and then incubated with 0–1.0 mM MU with or without exogenous 200 *μ*g ml^–1^ of HA for 72 h. Cultured cells and excised local tumours were subjected to HABP staining. Then, the cells and tissues were incubated with a 2.0 *μ*g ml^–1^ biotinylated HABP (b-HABP) probe for 2 h at room temperature. Bound b-HABP was detected by the addition of streptavidin-peroxidase reagents (Nichirei, Tokyo, Japan) and diaminobenzidine-containing substrate solution (Nichirei). As previously reported, both extracellular and cytoplasmic HA could be detected with bound b-HABP without permeabilisaton for cell staining (Nishida *et al*, 2005).

### Quantification of HA

The subconfluent LM8 cells were incubated with or without 1.0 mM MU for 6, 12, and 24 h. The isolation of HA was based on the methods reported by Tammi *et al* (2001). Briefly, the conditioned medium was collected and designated as ‘medium’. To remove the cell-surface-associated HA, the cells were incubated for 10 min at 37 °C with trypsin-EDTA and washed with PBS. The trypsin solution and combined washes were designated as ‘pericellular’. After cell counts, the cells were placed in Protease K solution (0.15 M Tris-HCl, pH 7.5, 0.15 M NaCl, 10 mM CaCl_2_, and 5 mM deferoxamine mesylate containing 20 units of protease K) and incubated for 2 h at 55 °C and the solution was designed as ‘intracellular’. All samples were heated at 100 °C for 15 min to inactivate protease activity and centrifuged at 15 000 g for 30 min at 4 °C, and the supernatants were analysed. The HA concentrations were measured using a sandwich enzyme-linked immunosorbent assay, as described previously (Zhu *et al*, 2010).

### Statistical analysis

All the *in vitro* quantitative experiments were performed more than 3 times, and analysis of variance followed by Bonferroni–Dunn *post-hoc* test was used to assess differences between means. Statistical comparisons between the two groups were made using an unpaired Student's *t-*test.

## Results

### MU inhibits HA accumulation in LM8, MG-63, and HOS cells

**Figure 1:**
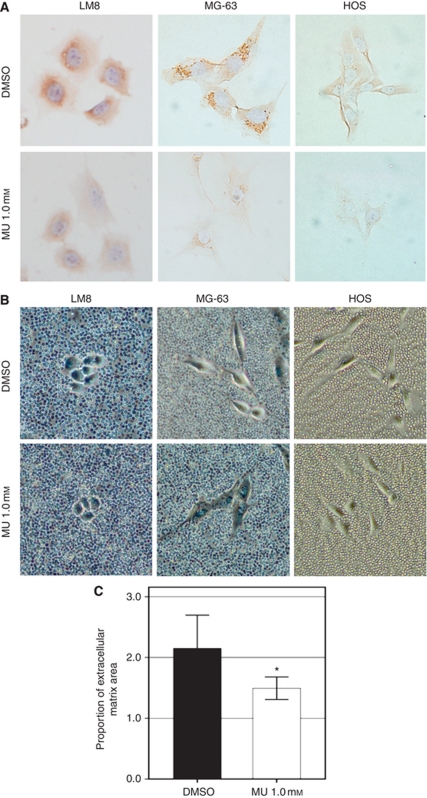
Effect of MU on HA accumulation in cells and pericellular matrix formation. (**A**) Histochemical staining of hyaluronic acid binding protein (HABP) in each cell that was either left untreated or incubated with MU (1.0 mM) for 72 h (original magnification × 200). (**B**) Visualised pericellular matrix that was observed after 72 h of incubation without or with MU (1.0 mM; original magnification × 200). (**C**) Morphometric analyses were used to determine the proportions of the area delineated by the cell-associated matrix area to the area delineated by the plasma membrane area. Bars represent means±s.d. from 10 cells of each condition (^*^P<0.001, compared with DMSO).

Cells treated with the control medium for 72 h showed prominent staining for HA. Cells treated with 1.0 mM MU for 72 h showed a substantial inhibition of HA staining (Figure 1A). Cells treated with 1.0 mM MU and 200 *μ*g ml^–1^ of exogenous HA also showed a faint staining, demonstrating no recovery in HA positivity in any cell line (data not shown). The amount of HA was measured per 10^5^ cells each in LM8 cells. The amount of pericellular HA treated with 1.0 mM MU for 6 h (0.405 ng) was significantly lower than that with control (DMSO) cells (0.514 ng; *P*<0.001). The amount of intracellular HA treated with 1.0 mM MU for 6 h (0.137 ng) was also significantly lower than that with control (DMSO) cells (0.195 ng; *P*<0.001). In contrast, the amount of HA in medium with MU treatment was not statistically different from that with control treatment. At 12, 24, and 48 h, the amount of HA with MU treatment tended to be lower than that with control treatment, but none of these differences reached statistical significance.

### MU inhibits cell-associated matrix formation

One of the functions of HA in tumour cells, particularly mesenchymal tumour cells, as well as other connective tissue cells such as chondrocytes, is to serve as a scaffold for the assembly of a cell-associated matrix (Knudson *et al*, 1996; Nishida *et al*, 1999). These matrices may favour independent cell growth, migration, and invasion. Tumour cells in the control medium displayed abundant cell-associated matrices after 72 h of culture (Figure 1B, particularly LM8 and MG-63 cells). However, MU treatment resulted in a substantial decrease in the diameter of cell-associated matrices (Figure 1B). Addition of exogenous HA (200 *μ*g ml^–1^) could not restore the cell-associated matrix formation in MU-treated LM8 cells (data not shown). Differences in functional cell-associated matrices in LM8 cells were determined by morphometric analysis (Figure 1C). The cell-associated matrix area in cells treated with MU was significantly lower than that in the control cells (*P*<0.001).

### MU alters Has and CD44 expression

**Figure 2:**
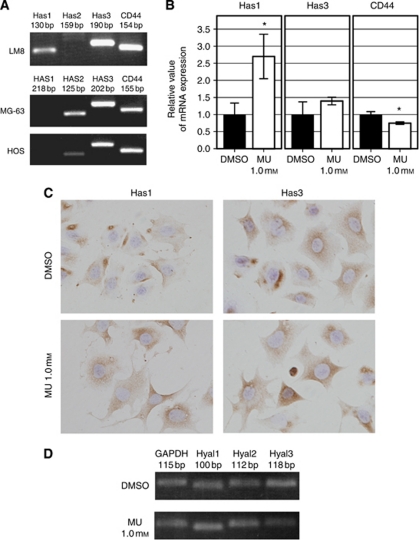
Effect of MU on hyaluronan and CD44 mRNA expression. (**A**) Conventional RT-PCR bands of hyaluronan synthase and CD44 transcripts. (**B**) The relative values of mRNA expression of Has1, Has3, and CD44 in LM8 cells were determined by real-time RT-PCR analysis. The data presented are the average±s.d. of relative mRNA expression values standardised by GAPDH mRNA expression. Differences between DMSO and MU were significant in Has1 and CD44, but not in Has3 (^*^P<0.05, compared with DMSO). (**C**) Immunocytochemical staining of Has1 and Has3 in LM8 cells that were treated with or without MU (1.0 mM) for 24 h (original magnification × 200). (**D**) Conventional RT-PCR bands of hyaluronidase in LM8 cells that were either left untreated or incubated with MU (1.0 mM) for 24 h.

The results of conventional RT-PCR are shown in Figure 2A. The reliability of the primer of Has2 and HAS1 was confirmed by the positive control cells: Has2 for Lewis lung cell carcinoma cell line, and HAS1 for MDA-MB-231 breast cancer cell line (data not shown). Although a previous study reported that of the three HA synthases, only Has3 was expressed in LM8 cells (Tofuku *et al*, 2006), both Has1 and Has3 mRNA could be detected. MG-63 cells expressed HAS2 and HAS3, but not HAS1. HOS cells expressed HAS2 and HAS3 mRNA. CD44, which is one of major cell-surface receptors of HA, was detected in each cell line. Results of real-time RT-PCR revealed that the treatment of LM8 cells with 1.0 mM MU for 24 h resulted in significant upregulation of Has1 (*P*=0.016) (Has3; *P*=0.152), whereas CD44 mRNA expression was downregulated (*P*=0.01, Figure 2B). The conventional RT-PCR revealed no marked change in the mRNA expression of HA degradation enzymes, Hyal1, Hyal2, and Hyal3, with or without treatment of 1.0 mM MU for 24 h (Figure 2D).

Results of immunocytochemistry for Has1 and Has3 showed more diffuse positive staining for Has1 in cells treated with MU than that in control cells. In contrast, there seemed to be no difference in Has3 staining between MU-treated cells and control cells (Figure 2C).

### MU inhibits cell proliferation and induces apoptosis

**Figure 3:**
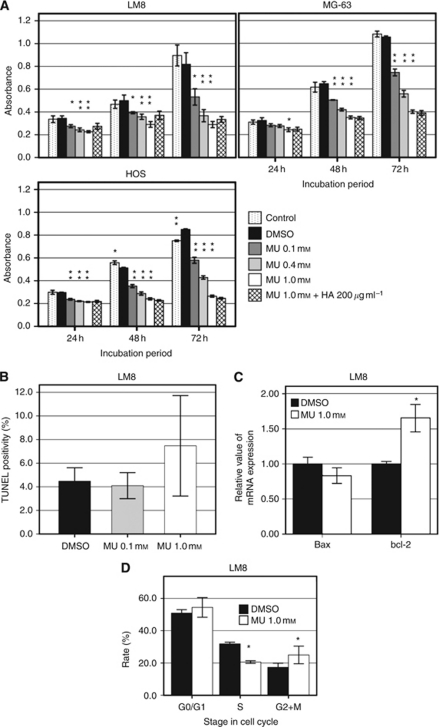
Effect of MU on cell proliferation, apoptotic activity, and cell cycles. (**A**) MTT assay. The cells were incubated with various concentrations of MU (dissolved in 1% DMSO) for 24, 48, or 72 h. The exogenous HA (∼8 × 10^5^ Da, 200 *μ*g ml^–1^) was added concurrently with MU. Bars represent means ±s.d. of absorbance readings at 550 nm from experiments performed in triplicate (^*^P<0.05, ^**^P<0.001, compared with DMSO; MU 1.0 mM+HA 200 *μ*g ml^–1^ group was compared with MU 1.0 mM group). (**B**) TUNEL staining. LM8 cells were cultured with or without MU for 48 h and then assayed by TUNEL staining. The percentage of positive cells with brown-stained nuclei was calculated in 20 different fields by using light microscopy at a magnification of × 200. Bars represent means±s.d. (**C**) The relative values of mRNA expression of Bax and bcl-2 in LM8 cells after treatment with or without MU for 24 h were determined by real-time RT-PCR analysis. The data presented are the average ±s.d. of relative mRNA expression values standardised by GAPDH mRNA expression (^*^P<0.05, compared with DMSO). (**D**) Changes in cell cycles were analysed by using CycleTEST PLUS DNA reagent kit. LM8 cells were incubated with or without 1.0 mM MU for 24 h and then evaluated by flow cytometry. (^*^P<0.05, compared with DMSO at the same stage on cell cycle).

As shown in Figure 3A, MU inhibited the proliferation of LM8, MG-63, and HOS cells in a dose-dependent manner. The proliferation of each cell treated with 1.0 mM MU for 24 h was lower (25–34% reduction) than that in DMSO (*P*<0.001 in LM8 and HOS cells and *P*=0.002 in MG-63 cells). Greater inhibition was observed after treatment with 1.0 mM MU for 48 h (42–53% reduction; *P*<0.001) and 72 h (62–69% reduction, *P*<0.001 in each line). However, exogenous HA added concurrently with MU did not cancel the effect of MU in any cell line. In MTT assay, no cytotoxicity of 1% DMSO on cell proliferation was observed as compared with the control cells (without DMSO). To determine the mechanism of the MU-mediated growth inhibition of LM8 cells, the number of apoptotic cells was determined by TUNEL staining. The average percentage of apoptotic cells treated with 1.0 mM MU was higher (1.67-fold) than that of the control. However, the difference between the values for control and the 1.0 mM MU treatment groups did not reach statistical significance (Figure 3B, *P*=0.288). Real-time RT-PCR for Bax and bcl-2 also revealed increased apoptotic activity with MU (Bax; *P*=0.119, bcl-2; *P*=0.025) expression for 12 h (Figure 3C).

### Effects of MU on cell cycle

Among the cells treated with 1.0 mM MU for 24 h, the number of cells in the S phase was significantly lower than the corresponding value for the control cells (*P*<0.05; Figure 3D), and the number of cells in the G2/M phases in cultures treated with 1.0 mM MU was significantly higher than the corresponding value for the control cells (*P*<0.05).

### MU inhibits cell migration and invasiveness

**Figure 4:**
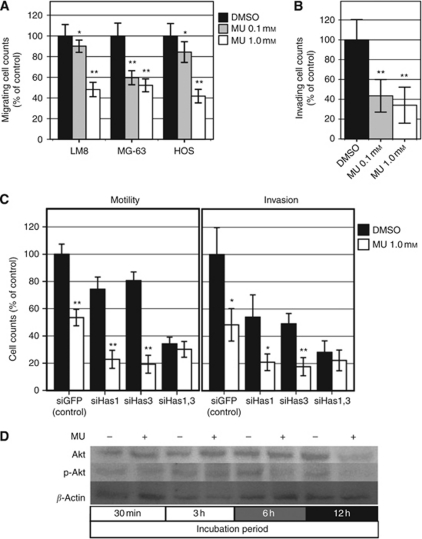
Effect of MU on cell motility, invasiveness, and Akt-phosphorylation. (**A**) Cell motility of LM8, MG-63, and HOS cells. (**B**) Cell invasiveness of LM8 cells. The number of cells on the lower surface of the membrane was counted in 20 randomly selected high-power fields. The data are presented as the average±s.d. (^*^P<0.05, ^**^P<0.001, compared with DMSO). (**C**) Cell motility and invasiveness of LM8 cells after knockdown of Has1 and/or Has3. The data are presented as the average±s.d. (^*^P<0.05, ^**^P<0.001, compared with DMSO). (**D**) Western blotting for Akt phosphorylation.

At the 24-h time point, the migratory activity of LM8, MG-63, and HOS cells treated with 1.0 mM MU was significantly lower (LM8; 52% reduction, MG-63; 48%, and HOS; 58%) than that of the control cells (*P*<0.001, in each cell line, Figure 4A), whereas the migratory activity of cells treated with 0.1 mM MU varied between cell lines (LM8: 10% reduction, *P*=0.037; MG-63: 40% reduction, *P*<0.001; HOS: 16% reduction, *P*=0.004). In the invasion assay, the capacity of the MU-treated LM8 cells to pass though the Matrigel-coated filters was significantly lower than that of the control cells (Figure 4B). The invasiveness of cells treated with 0.1 and 1.0 mM MU was 57% (*P*<0.001) and 66% (*P*<0.001), respectively, lower than that of the control. Under the condition of Has1 and/or Has3 knockdown with siRNA (efficiency: single knockdown of Has1, 95% Has3, 92% double knockdown of Has1 and Has3, 95% and 73%, respectively), the single knockdown of Has1 or Has3 did not compensate for the effects of MU on cell motility or invasiveness, whereas the double knockdown of Has1 and Has3 did (Figure 4C). These results suggested that the inhibitory effects of MU on cell motility and invasiveness are mediated via an HA-dependent route.

### MU inhibits Akt phosphorylation

A previous report showed the important roles of Akt signalling in the development of pulmonary metastases of osteosarcoma by using LM8 cells (Fukaya *et al*, 2005). Another study using carcinoma cells showed that perturbation of HA-CD44 binding leads to suppression of the phosphoinositide 3-kinase (PI3K)/Akt pathway, thereby inhibiting cell and tumour growth (Ghatak *et al*, 2002). We examined Akt phosphorylation after MU treatment. Western blot analysis showed that Akt phosphorylation in cells treated with MU at 6 and 12 h was lower than that in the control cells, although no difference was observed at 30 min or 3 h (Figure 4D).

### MU had inhibitory effects on local tumour growth and lung metastases

**Figure 5:**
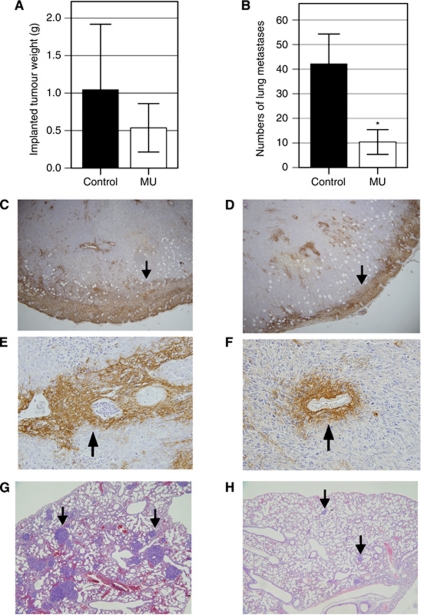
Effect of MU on implanted tumour mass and lung metastasis of LM8 cells. (**A**) The wet weights of the transplanted tumour were measured (difference was not significant). (**B**) The numbers of lung metastases at the coronal midline section. Representative sections of transplanted tumours with HABP staining (**C** and **E**; Control, **D** and **F**; MU treatment, original magnification × 40; **E**). Representative sections of the lung are shown (**G**, Control; **H**, MU treatment; original magnification × 40). Bars represent means±s.d. (^*^P<0.05, compared with control).

Daily administration of MU showed an inhibitory effect on LM8 tumour growth, based on a reduction in tumour wet weight (49% reduction, Figure 5A). However, because of the deviations between values for different tumours, the overall values did not reach significance (*P*=0.251). The inhibitory effects of MU treatment on local tumour growth may be attributable to decreased HA retention by LM8 cells and/or stromal cells. Analysis of HA by HABP staining revealed that HA retention in MU-treated LM8 local tumours (Figure 5D and F) was lower than that in the control tumours (Figure 5C and E). Interestingly, HA retention was notable in the periphery of control tumours (Figure 5C); however, retention was prominently suppressed by MU treatment (Figure 5D). In addition, HA retention in the surrounding stromal tissues and perivascular lesions with MU treatment was also lower (Figure 5F) than that in the control (Figure 5E). Treatment with MU resulted in a significant (75%) reduction in the number of metastatic lung lesions (*P*=0.009, Figure 5B). These results can be compared visually. The number of lung metastases in MU-treated mice (Figure 5H) was lower than that in control mice (Figure 5G).

## Discussion

In this study, we hypothesised that the inhibition of HA synthesis by MU in osteosarcoma cells might decrease the formation of cell-associated matrices, thereby leading to the suppression of tumourigenicity and of metastasis. We showed that MU exerted a multistep inhibitory effect on the tumourigenicity of osteosarcoma cells via inhibition of HA synthesis. Previous reports indicated that at least two steps in HA synthesis are affected by MU. First, MU depletes cellular UDP-GlcUA, which is a precursor of HA, because of massive glucuronic acid conjugation to MU (Kakizaki *et al*, 2004; Kultti *et al*, 2009). Second, mRNA levels of HASs are downregulated by MU, resulting in the suppression of HA synthesis. A dose-dependent reduction in the mRNA levels of HAS2 or HAS3 was observed in all the cancer cell lines examined (Kultti *et al*, 2009), which is not consistent with the findings of the current study, in which Has1 and Has3 mRNA levels increased after treatment with MU. A possible explanation for the results of our study is that the reduction of HA synthesis induced by UGA depletion by MU causes positive feedback for Has mRNA expression to compensate for the decreased deposition of HA. In addition, we speculate that the alteration of Has expression by MU depends on cell lines or incubation periods. Although transcriptional repression of the Has mRNAs is apparent, MU-initiated cellular signalling pathways have not been known to affect Has expression. 4-Methylumbelliferone had no significant impact on mRNA expression of CD44, a principal cell-surface receptor of HA. This result is in agreement with previous reports on other cell types, in which MU did not alter CD44 mRNA (Rilla *et al*, 2004; Kultti *et al*, 2009). In the osteosarcoma cell line LM8, the suppression of Has by MU may be dominated not by the downregulation of Has mRNA but by depletion of UGA.

Cell proliferation was suppressed by MU administration, and the same inhibitory effects were reported in studies with pancreatic cancer cells (Nakazawa *et al*, 2006), melanoma cells, and breast cancer cells (Kultti *et al*, 2009). The same inhibitory effects on cell motility and invasion demonstrated in the current study were also reported in studies on melanoma cells, breast cancer cells (Kultti *et al*, 2009), and prostate cancer cells (Lokeshwar *et al*, 2010); however, these studies investigated the effects of MU primarily on malignant cells of epithelial origin. The behaviour of these cells may differ from that of malignant tumours of mesenchymal origin. Previous studies revealed that many osteosarcoma cells have abundant HA-rich cell-associated matrices (Nishida *et al*, 2005; Hosono *et al*, 2007; Suzuki *et al*, 2009). Although previous reports described the cell-associated matrix formation in melanoma cells (Kudo *et al*, 2004) and pancreatic cancer cells (Nakazawa *et al*, 2006), the functional cell-associated matrices observed by particle exclusion assay were smaller than those observed with osteosarcoma cells (Nishida *et al*, 2005; Hosono *et al*, 2007; Suzuki *et al*, 2009).

As HA is a major component of ECM, the reduction of HAS or Has subsequently causes the suppression of ECM production, particularly that of the cell-associated matrix. Several previous studies addressed the relationship between cell-associated matrix and tumourigenicity (Nishida *et al*, 2005; Hosono *et al*, 2007; Monz *et al*, 2008). In these studies, the inhibition of the cell-associated matrix formation by genetic manipulation of HAS or use of exogenous HA oligosaccharides resulted in reduced tumourigenicity. In the present study, the inhibition of cell-associated matrix formation via suppression of HA synthesis by MU effectively suppressed the tumourigenicity. Thus, the efficacy of MU for anti-tumour activity may be partly because of the depletion of cell-associated matrix formation, although other mechanisms may also inhibit the tumourigenicity induced by MU, such as a reduction of HA in cytoplasm (Nishida *et al*, 2005). Further studies will be required to clarify the precise mechanisms of the anti-tumour effects of MU.

In the present study, addition of exogenous HA did not cancel the effect of MU on HA accumulation in cells, formation of cell-associated matrix, or cell proliferation. We speculate that differences in biological potency between exogenous HA (free HA) and endogenous HA (cell-associated HA) may exist.

Although HA-CD44 signalling is known to result in the stimulation of cell motility and proliferation (Kim *et al*, 2005), the molecular mechanisms of this HA-induced enhanced tumourigenicity remain poorly understood. Recent studies have shown that the PI3K/Akt signalling pathway is significantly involved in HA-induced cell motility and invasiveness. For example, in HA-mediated breast cancer progression, the binding of HA to MDA-MB-231 cells induces PI3K/Akt activation, which is effectively blocked by a PI3K inhibitor (Bourguignon *et al*, 2003; Kim *et al*, 2005). Exogenous HA oligosaccharides blocked endogenous HA binding to CD44, resulting in the inhibition of PI3K/Akt signalling pathway in murine mammary carcinoma cells (Ghatak *et al*, 2002). Thus, exogenously administered agents have been well analysed, whereas very few studies have investigated whether MU, which inhibits endogenous HA synthesis, affects the PI3K/Akt pathway. In prostate cancer cells, MU induced downregulation of phosphorylated Akt, indicating that Akt signalling is an important mechanism in the antitumour activity of MU (Lokeshwar *et al*, 2010). This study, for the first time, demonstrated MU-induced downregulation of Akt phosphorylation in osteosarcoma cells. Considering the delayed inhibition of Akt phosphorylation (after 6 h) by MU in this study, MU may indirectly affect Akt phosphorylation, possibly via suppression of HA synthesis, perturbation of HA-receptor interaction, or alteration of cell signalling pathways including Akt phosphorylation.

The degree of the inhibitory effects of MU on the formation of lung metastasis *in vivo* was markedly higher than that on the growth of the implanted primary tumour. In contrast to the growth of the primary tumour, multistep processes are associated with distant metastasis. In this study, MU suppressed proliferation, motility, and invasion of osteosarcoma cells *in vitro*. Inhibition of these steps by MU led to substantial suppression of lung metastasis. Another explanation is that MU affects the microenvironment of the primary and target organs. The tumour stroma and surrounding normal cells (immune cells, inflammatory cells, pericytes, vascular endothelial cells, and fibroblasts) can be affected by MU, possibly via suppression of HA synthesis. Notably, in the current study, HA deposits were markedly suppressed not only in the periphery of the tumour, but also in the surrounding stromal tissues and perivascular region *in vivo.* In the clinical context, the strong suppressive effects of MU on lung metastasis might be especially beneficial for patients with osteosarcoma, considering that the primary cause of death in this group is lung metastasis (Ta *et al*, 2009).

Several studies have shown suppression of tumourigenicity by using genetic modification of HASs or the administration of HA oligos (Simpson *et al*, 2002; Nishida *et al*, 2005; Hosono *et al*, 2007). However, genetic manipulation has limited clinical applicability because of the predicted complications and ethical issues, and the administration of HA oligos is challenging in terms of drug delivery. In contrast, as MU has already been clinically used as an oral choleretic agent in Japan, it can be readily used for osteosarcoma patients. However, the optimal dose to attain anticancer effects needs to be established. In the United States, the oral administration of MU at 2.2 g day^–1^ has been used in trials for the treatment of hepatitis B and C (ClinicalTrials.gov. number, NCT00225537). The complications and dose-limiting toxicity of MU treatment will be clarified after clinical trials.

In conclusion, we showed that MU inhibited various processes of tumourigenicity *in vitro* in murine and human osteosarcoma cell lines, and markedly suppressed lung metastasis in highly metastatic murine osteosarcoma cells. Although clinical trials are required to clarify the efficacy of MU in human patients with osteosarcoma, the results of this study suggest that MU might be a novel antimetastasis agent for the treatment of osteosarcoma.
