# Supplementary material for: Inhibition of hyaluronan retention by 4-methylumbelliferone suppresses osteosarcoma cells in vitro and lung metastasis in vivo
Source: Br J Cancer. 2011 Nov 1;105(12):1839–49. doi: 10.1038/bjc.2011.459 (PMC3251882; doi:10.1038/bjc.2011.459)
Supplement: Supplementary Information [file bjc2011459x1.doc]

**Supplemental online material**

The Has 1, Has 2, Has 3, murine CD44, Hyal1, Hyal2, Hyal3, Bax, bcl-2 and murine GAPDH primer pairs were as follows: Has 1 sense, 5′-GGCACCCACTGCACATTT-3′; Has 1 antisense, 5′-TCAACCAACGAAAGGAG-3′ (predicted PCR product of 130 bp); Has 2 sense, 5′-TGGGGTGGAAAGAGAGAA-3′; Has 2 antisense, 5′-TCCAACACCTCCAACCAT-3′ (predicted PCR product of 159 bp); Has3 sense, 5′-CAGCACCTTCTCATGCATCAT-3′; Has3 antisense, 5′-TCCTCCAACACCTCCTACTTG-3′ (predicted PCR product of 190 bp); murine CD44 sense, 5′-GAGGATTCATCCCAACGCTA-3′; murine CD44 antisense, 5′-GAAGGAATTGGGTAGGTCTGTG-3′ (predicted PCR product of 154 bp); Hyal1 sense, 5'-CAGACAAAACAAGTACCAAGGA-3'; Hyal1 antisense, 5'-AAAAGAGCTGCACTGGTCA-3' (predicted PCR product of 100bp); Hyal2 sense, 5'-AGCCAGGTGGACCTTATC-3'; Hyal2 antisense, 5'-GGTATTGGCAGGTCTCCAT-3' (predicted PCR product of 112bp); Hyal3 sense, 5'-GTCTCTGGACGACCTGATG-3'; Hyal3 antisense, 5'-ATGGAGACGCCAGCACTT-3' (predicted PCR product of 118bp); Bax sense, 5'-CCGGCGAATTGGAGATGAAC-3'; Bax antisense, 5'-ACAGGGCCTTGAGCACCAGT-3' (predicted PCR product of 188bp); bcl-2 sense, 5'-TTCCAGCCTGAGAGCAAC-3'; bcl-2 antisense, 5'-CATCTCTGCGAAGTCACGA-3' (predicted PCR product of 186bp); murine GAPDH sense, 5′-AAATGGTGAAGGTCGGTGTG-3′; murine GAPDH antisense, 5′-AGGTCAATGAAGGGGTCGTT-3′ (predicted PCR product of 115 bp). The HAS 1, HAS 2, HAS 3, human CD44, and human GAPDH primer pairs were as follows: HAS1 sense; 5'-CAGACCCACTGCGATGAGAC-3', HAS1 antisense; 5'-CCACCAGGTGCGCTGAAA-3' (predicted PCR product of 218bp), HAS2 sense; 5'-TCAGAGCACTGGGACGAAG-3', HAS2 antisense; 5'-CCCAACACCTCCAACCAT-3' (predicted PCR product of 125bp), HAS3 sense; 5'-CAGCAACTTCCATGAGGC-3', HAS3 antisense; 5'-CACAGTGTCAGAGTCGCA-3' (predicted PCR product of 202bp), CD44 sense; 5'-TTGCAGTCAACAGTCGAA-3', CD44 antisense; 5'-TTCTGACGACTCCTTGTTC-3' (predicted PCR product of 155bp), GAPDH sense; 5'-TGAACGGGAAGCTCACTGG-3', antisense; 5'-TCCACCACCCTGTTGCTGTA-3' (predicted PCR product of 307bp).
